# Supplementary material for: Improved prediction of smoking status via isoform-aware RNA-seq deep learning models
Source: PLoS Comput Biol. 2021 Oct 11;17(10):e1009433. doi: 10.1371/journal.pcbi.1009433 (PMC8530282; doi:10.1371/journal.pcbi.1009433)
Supplement: S1 Text — Supplemental experiment results on upper-quartile normalized dataset and results after removing the 5 genes from the Beineke model and correlated gene partners. S1A Table: Predictive performance of modified Beineke models using gene, isoform and exon-level expression data, with MUC1 included and upper-quartile normalized. Val: validation data. AUC: area under the receiver operating characteristic. IML-GTF: Isoform Map Layer containing information from Ensembl GTF file. FSL: Feature Selection Layer. Best results are shown in bold. S1B Table: Predictive performance of modified Beineke models using gene, isoform and exon-level expression data, with MUC1 included and TMM normalized as in the main paper. Val: validation data. AUC: area under the receiver operating characteristic. IML-GTF: Isoform Map Layer containing information from Ensembl GTF file. FSL: Feature Selection Layer. Best results are shown in bold. S1C Table: Predictive performance of various deep learning models using exon-level data processed with upper-quartile normalization. Val: validation data. AUC: area under the receiver operating characteristic. IML-GTF: Isoform Map Layer containing information from Ensembl GTF file. FSL: Feature Selection Layer. Best results are shown in bold. S1D Table: Predictive performance of deep learning models using all 1079 genes, and 1020 genes with genes used in the Beineke model and their correlated genes (correlation coefficient ≥ 0.4) removed, and TMM normalized as in the main paper. Val: validation data. AUC: area under the receiver operating characteristic. Base: the base architecture for gene input, Input-128-64-32-Output. Best results are shown in bold. S1E Table: Predictive performance of modified Beineke models using gene, isoform and exon-level expression data, with MUC1 included and covariate signals removed from the data. Val: validation data. AUC: area under the receiver operating characteristic. IML-GTF: Isoform Map Layer containing information from Ensembl [file pcbi.1009433.s001.pdf]

Supplemental results

Results on upper-quartile normalized dataset

We renormalized our data using upper-quartile normalization [1] separately in the training and testing data to ensure no leakage of information through the normalization procedure, and we repeated all of our analyses in this re-normalized data. As can be seen in S1A and S1C Tables, our results are consistent using both TMM and upper-quartile normalization, indicating that the performance of our models is robust to use of a different normalization approach that is not based on the selection of a single reference sample.

Despite the low level expression of *MUC1*, we added *MUC1* to the set of genes and the corresponding isoform and exon features for completeness. The results in S1A Table are similar to the results shown in our main text for all exon based models.

Results after removing the 5 genes from the Beineke model and correlated gene partners

To assess the importance of the genes used in the Beineke model, we remove these genes together with their correlated genes (with correlation coefficient ≥ 0.4) from the larger feature set of 1079 genes, resulting in 1020 genes. We use the base deep learning model for gene input. The final result is shown in S1D Table. Comparing the test AUC (0.900 versus 0.856), we can see that removing these genes and their correlated partners indeed results in a decrease in performance. However, based on the information contained within the remaining uncorrelated genes, our model is able to give reasonable predictive results.

Supplemental Tables 23

S1A Table. Predictive performance of modified Beineke models using gene, isoform and exon-level expression data, with *MUC1* included and upper-quartile normalized.

	Val - Accuracy	Val - AUC	Test - Accuracy	Test - AUC
Gene	0.673	0.687	0.698	0.752
Isoform	0.781	0.807	0.803	0.794
Exon	0.808	0.844	0.808	0.869
Exon, IML-GTF	**0.828**	**0.874**	**0.840**	**0.871**
Exon, IML-GTF, FSL	0.818	0.863	0.813	0.869

Val: validation data. AUC: area under the receiver operating characteristic. IML-GTF: Isoform Map Layer containing information from Ensembl GTF file. FSL: Feature Selection Layer. Best results are shown in bold.

S1B Table. Predictive performance of modified Beineke models using gene, isoform and exon-level expression data, with *MUC1* included and TMM normalized as in the main paper.

	Val - Accuracy	Val - AUC	Test - Accuracy	Test - AUC
Gene	0.668	0.651	0.669	0.666
Isoform	0.752	0.813	0.758	0.815
Exon	0.806	0.86	0.782	0.855
Exon, IML-GTF	**0.830**	0.876	0.821	0.871
Exon, IML-GTF, FSL	**0.830**	**0.892**	**0.834**	**0.875**

Val: validation data. AUC: area under the receiver operating characteristic. IML-GTF: Isoform Map Layer containing information from Ensembl GTF file. FSL: Feature Selection Layer. Best results are shown in bold.

S1C Table. Predictive performance of various deep learning models using exon-level data processed with upper-quartile normalization.

	Val - Accuracy	Val - AUC	Test - Accuracy	Test - AUC
Exon Base	0.832	0.895	0.854	0.909
Exon, IML-GTF	0.847	0.916	0.856	0.918
Exon, IML-GTF, FSL	**0.858**	**0.916**	**0.858**	**0.936**

Val: validation data. AUC: area under the receiver operating characteristic. IML-GTF: Isoform Map Layer containing information from Ensembl GTF file. FSL: Feature Selection Layer. Best results are shown in bold.

S1D Table. Predictive performance of deep learning models using all 1079 genes, and 1020 genes with genes used in the Beineke model and their correlated genes (correlation coefficient ≥ 0.4) removed, and TMM normalized as in the main paper.

	Val - Accuracy	Val - AUC	Test - Accuracy	Test - AUC
1079 Gene, Base	**0.828**	**0.900**	**0.830**	**0.900**
1020 Gene Uncorrelated, Base	0.788	0.825	0.782	0.856

Val: validation data. AUC: area under the receiver operating characteristic. Base: the base architecture for gene input, Input-128-64-32-Output. Best results are shown in bold.

S1E Table. Predictive performance of modified Beineke models using gene, isoform and exon-level expression data, with *MUC1* included and covariate signals removed from the data.

	Val - Accuracy	Val - AUC	Test - Accuracy	Test - AUC
Gene	0.675	0.677	0.688	0.713
Isoform	0.788	0.845	0.743	0.806
Exon	0.808	0.866	0.766	0.842
Exon, IML-GTF	**0.835**	0.868	0.797	0.836
Exon, IML-GTF, FSL	0.818	**0.869**	**0.823**	**0.867**

Val: validation data. AUC: area under the receiver operating characteristic. IML-GTF: Isoform Map Layer containing information from Ensembl GTF file. FSL: Feature Selection Layer. Covariates removed: age, sex, body-mass index, and batch. Best results are shown in bold.

S1F Table. Predictive performance of various deep learning models using the full set of exon-level data, with covariates signals removed from the data.

	Val - Accuracy	Val - AUC	Test - Accuracy	Test - AUC
Exon Base	0.830	0.884	0.832	0.901
Exon, IML-GTF	**0.867**	0.921	0.834	0.900
Exon, IML-GTF, FSL	0.867	**0.933**	**0.846**	**0.905**

Val: validation data. AUC: area under the receiver operating characteristic. IML-GTF: Isoform Map Layer containing information from Ensembl GTF file. FSL: Feature Selection Layer. Covariates removed: age, sex, body-mass index, and batch. Best results are shown in bold.

S1G Table. Institutional review board approval protocol numbers. IRB protocol numbers at all participating COPDGene study sites are included as a separate Word document.

References

1. Bullard JH, Purdom E, Hansen KD, Dudoit S. Evaluation of statistical methods for normalization and differential expression in mRNA-Seq experiments. BMC bioinformatics. 2010;11(1):1–13.
