# Supplementary figures and images for: Improved prediction of smoking status via isoform-aware RNA-seq deep learning models
Source: PLoS Comput Biol. 2021 Oct 11;17(10):e1009433. doi: 10.1371/journal.pcbi.1009433 (PMC8530282; doi:10.1371/journal.pcbi.1009433)

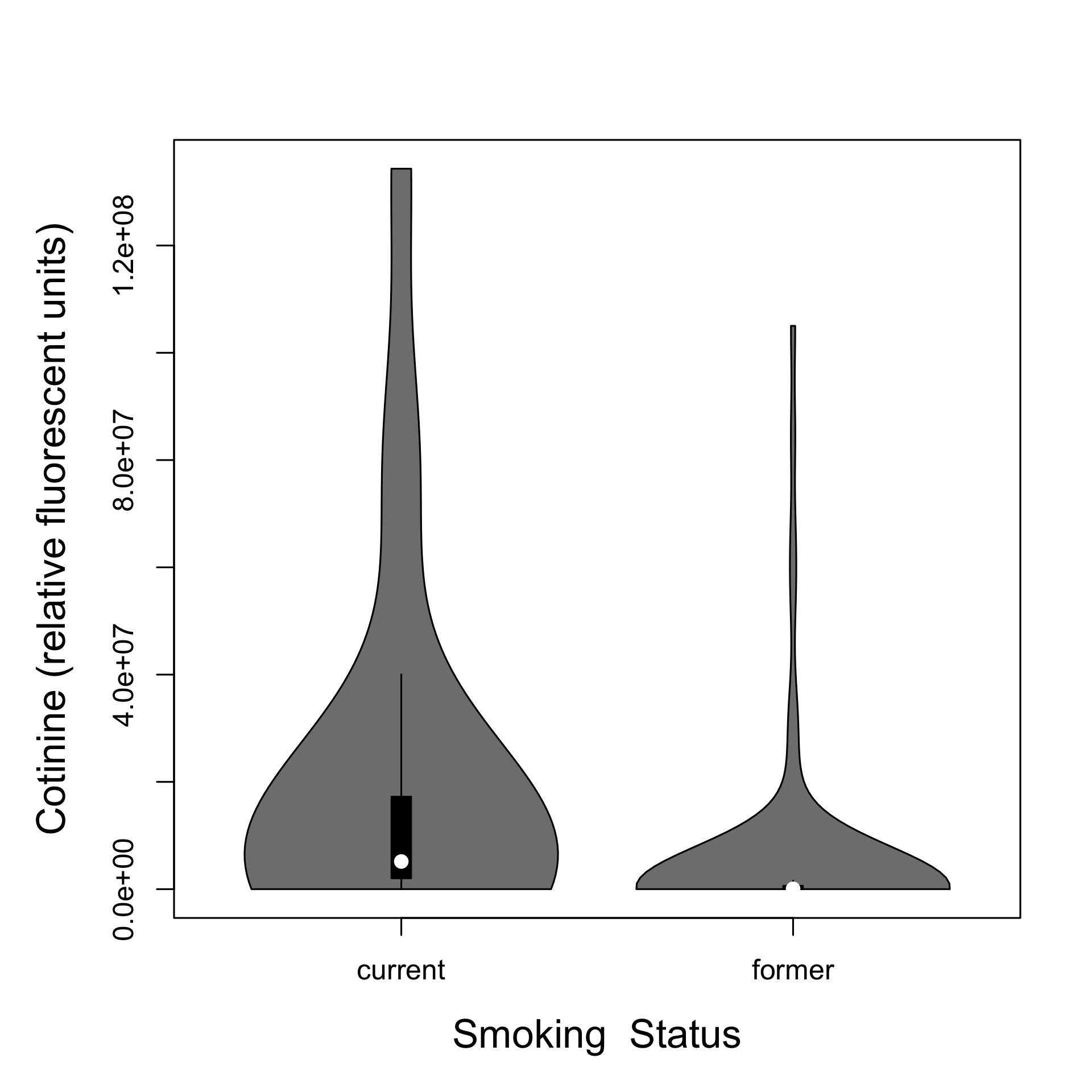

Supplement: S1 Fig — Plasma levels of cotinine are higher in current smokers (N = 21) compared to former smokers (N = 85) in subjects from COPDGene at the second study visit. (TIF) [file pcbi.1009433.s003.tif]

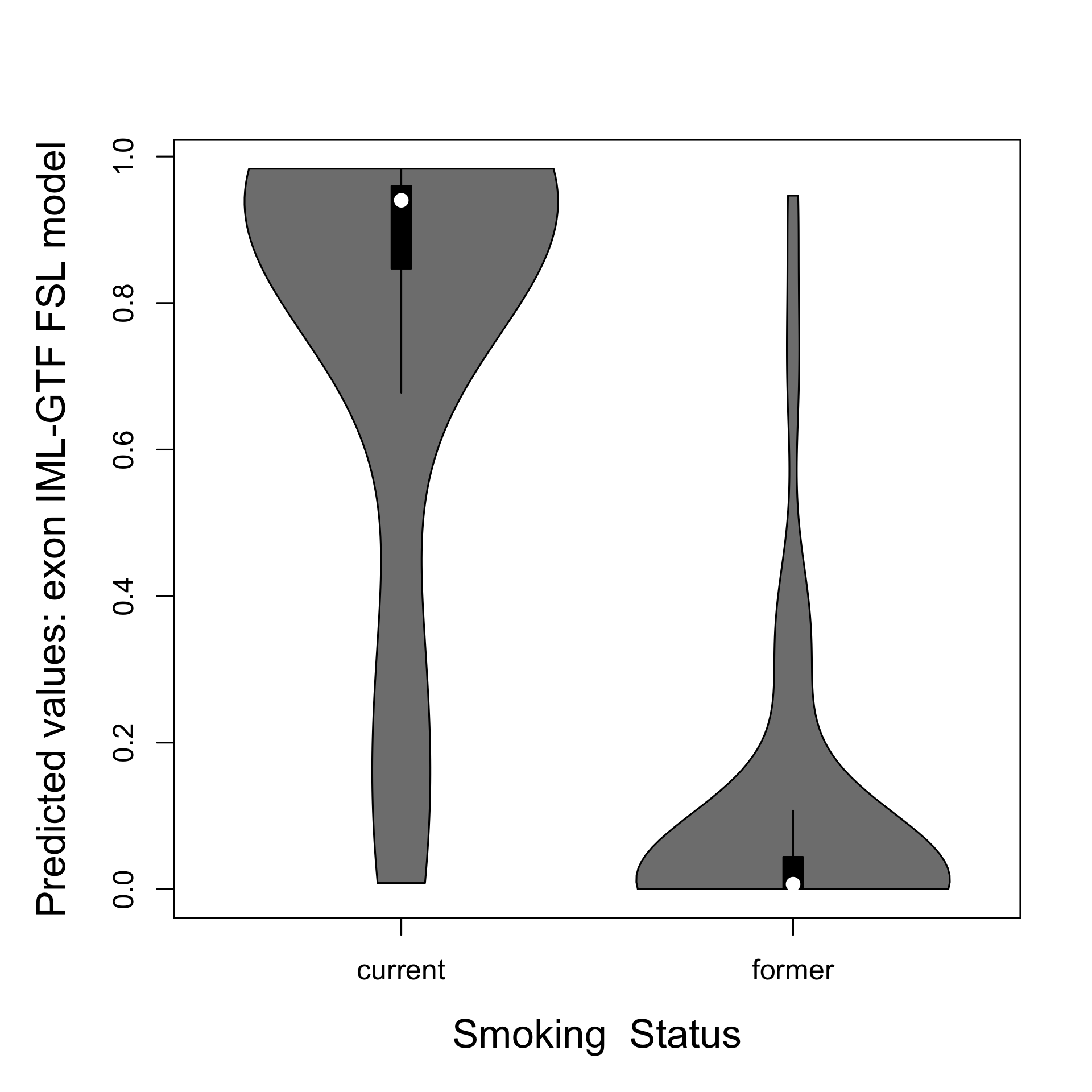

Supplement: S2 Fig — Predicted values from the exon model with the isoform map and feature selection layer are higher for current smokers (N = 21) than for former smokers (N = 85) in a subset of subjects from COPDGene with concurrent plasma cotinine values also available. (TIF) [file pcbi.1009433.s004.tif]

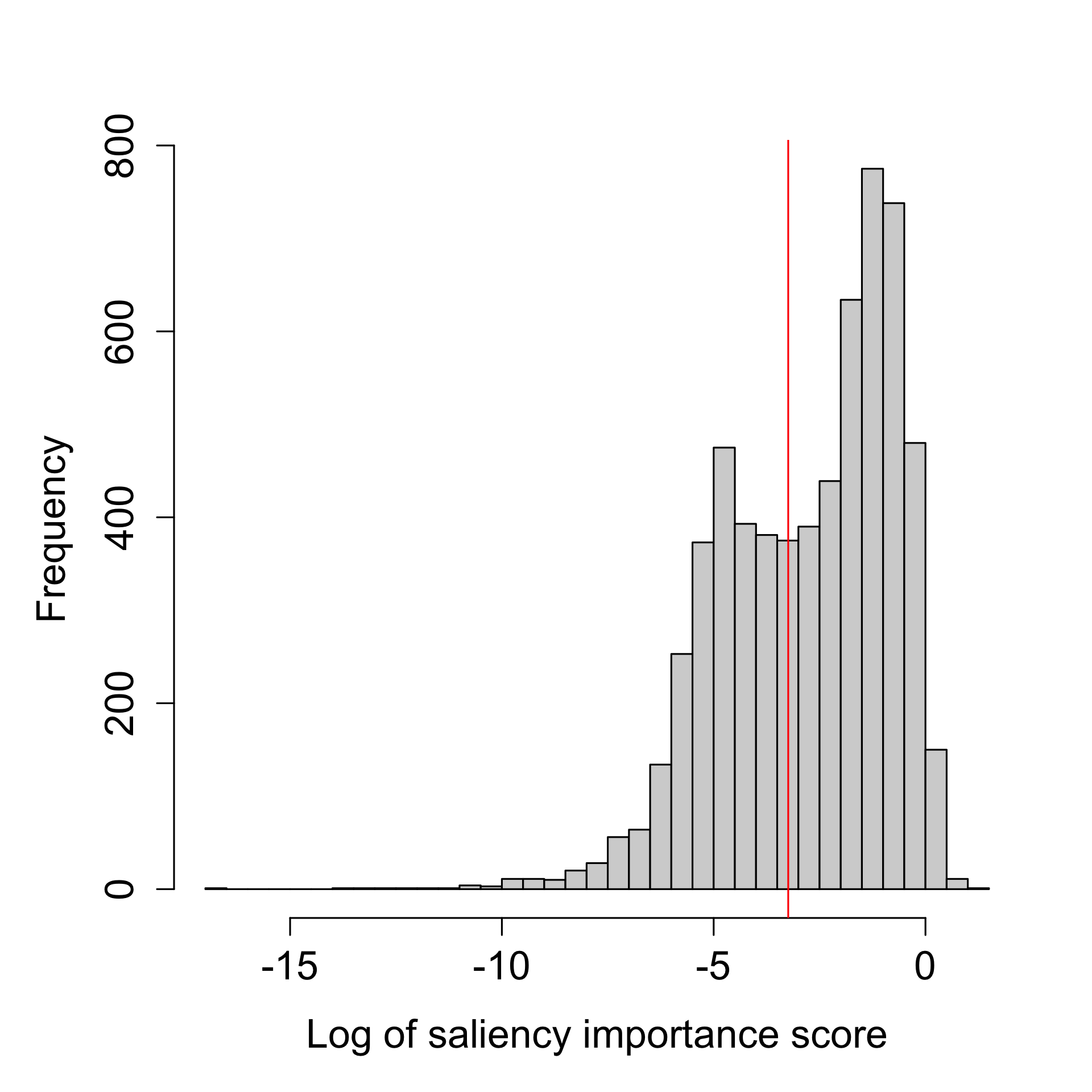

Supplement: S3 Fig — The distribution of log of feature importance scores for each exon using the DeepExplain [20] framework with saliency maps [21] on the trained Exon, IML-GTF, FSL model. 48.5% of exons had non-zero scores, and the distribution of the 243 non-zero scores was bimodal. The red line in the figure indicates the top 20% of exons. (TIF) [file pcbi.1009433.s005.tif]

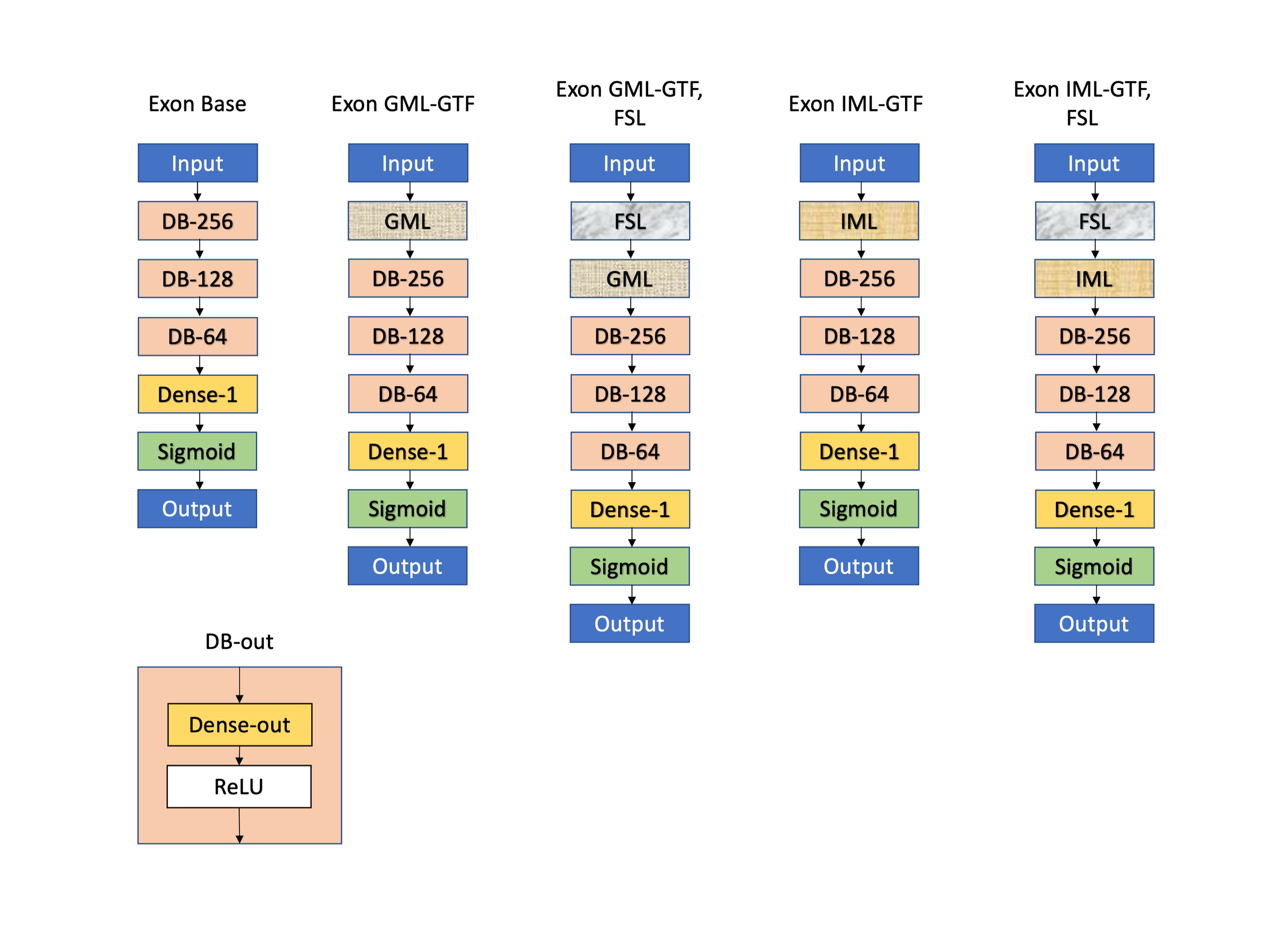

Supplement: S4 Fig — IML: Isoform Map Layer containing information from GTF file. GML: Gene Map Layer containing information from GTF file. FSL: Feature Selection Layer. (TIF) [file pcbi.1009433.s006.tif]
