# Supplementary material for: Improved prediction of smoking status via isoform-aware RNA-seq deep learning models
Source: PLoS Comput Biol. 2021 Oct 11;17(10):e1009433. doi: 10.1371/journal.pcbi.1009433 (PMC8530282; doi:10.1371/journal.pcbi.1009433)
Supplement: Attachment — Submitted filename: review_comments.pdf [file pcbi.1009433.s007.pdf]

General Opinion:

In the manuscript "Improved prediction of smoking status via isoform-aware RNA-seq deep learning models" from Wang et al., the authors investigate the task of predicting smoking status from gene expression data. For covariates, they use RNA-seq expression measurements at varying resolutions, including the abundances of whole-genes, specific isoforms, and even individual exons. For gene set selection, the authors use 1,270 differentially expressed genes from the COPDGene study. They also compare their model to a modified version of a previously published one from Beineke et al. that used five genes. The model using the full set of 1,270 genes performs much better than the one using only four genes (+0.06 testing AUROC). After showing the exon-level abundance covariates to be most performant, they improve (+0.017 validation AUROC for Beineke-based model, +0.001 testing AUC for Beineke-based model, +0.019 validation AUROC for full model, +0.011 testing AUROC for full model) on their models with a seemingly novel "isoform map layer" that maps individual exon covariates to isoform-specific ones that they then use to expand the set of input features for subsequent layers. They also explore the use of an L1-constrained "feature selection layer", a bijective mapping of input to output features via non-negative rescaling, which improves the model's performance as well (+0.013 validation AUROC for Beineke-based model, +0.005 testing AUROC for Beineke-based model, +0.011 validation AUROC for full model, +0.011 testing AUROC for full model). While this is an interesting paper with clear methodological contributions, it does have some weaknesses. In particular, the analysis of the final model is very limited, and the biological relevance could be improved. Lastly, I have concerns about how easy this paper will be to reproduce due to the absence of the code and other important resources.

Major Points:

1) The current introduction does little to frame the work's methodological contributions and innovations with respect to the existing literature on deep learning applications for computational biology. This is unfortunate due to the apparent novelty of the isoform map layer and other important contributions. Most of the introduction rehashes the authors' previous contributions to the field of transcriptomics analysis, or references specific biological mechanisms (e.g. T-cell activation) relevant to their data that are mentioned nowhere else in the paper. It would be appropriate to have at least one reference and sentence mentioning deep learning for computational biology in specific (e.g. one of the many reviews out there, such as dx.doi.org/10.1098/rsif.2017.0387 or another similar review). To a lesser extent, it may also be worth noting the several previous applications of deep learning to splicing and isoforms (e.g. most notably dx.doi.org/10.1126/science.1254806 and www.nature.com/articles/s41592-019-0351-9), although the focuses of such works have been different from this one's.
2) Although the transcriptomic data for the manuscript has been uploaded to GEO, I could not find the list of specific gene/exon/isoform covariates that were used in the models. While the list of 1,270 genes is

readily available in Huan et al., the others are not. The authors do give the version of ENSEMBL that they used, and some vague instructions on how to derive and filter the isoforms and exons. However, it would behoove the authors to also include the exon and isoform definitions themselves as supplementary data. At present the reproducibility of their entire paper hinges entirely on this point, as well as on Biomart's continued support and availability of old releases. Nevertheless, even if one acquires the correct GTF and performs the procedures in the methods, there is no way to verify that the resultant sets and definitions exactly match the ones used in the paper. Ideally, the authors would also include the code used to derive the exon definitions and reproduce their paper and archive it publicly (e.g. on Zenodo or elsewhere).

3) I could not find a full description of the network architecture used for each set of covariates. This is critical to understanding the paper, and it is incomplete without it. The authors have also left out the learned weights for their neural network model, as well as the code used for their model. These software artifacts are essential to reproducing and understanding this paper, since subtle implementation differences can lead to drastically different outcomes.
4) The discussion of cotinine and the model's applicability has a few issues. For instance, the authors mention that the model could be used in scenarios where transcriptomic data are available, but cotinine measurements are not. The authors also state that their model performs worse than cotinine measurements for classifying smoking status. However, this is merely assumed based on cotinine's performance as a predictor on entirely different datasets. It would be more accurate for the authors to instead state that cotinine measurements are known to be a strong predictor of smoking status, but it is unknown how their model will compare to them. Clearly, this is not ideal. Thus, if there exists a dataset of paired cotinine and RNA-seq expression data, then evaluation on said dataset with the author's model seems like a needed addition.
5) It appears that they normalized their training/validation/testing data using the trimmed mean of M values (TMM) implementation in the edgeR library. However, since the authors have not included their source code, it is not immediately obvious which samples were chosen as reference samples for the normalization step. This turns out to be critical. If the reference sample was included in the validation or testing data, then it represents a leakage of test set information and could lead to inflated test performance estimates. It is entirely possible that this is not the case however. Hopefully, the authors can clear up this confusion. In general, an evaluation on additional RNA-seq data from another cohort would more convincingly demonstrate the model's ability to generalize beyond the COPDGene cohort and RNA-seq batches.
6) Does inclusion of *MUC1* have a significant effect on the model performance? I realize that it has a low abundance, but that does not necessarily mean that it is irrelevant or would not influence the model performance in a significant way. At present, since *MUC1* was not included in their Beineke-based

model, it does not seem like there has been a proper evaluation of the original Beineke model. Along those lines, are any or all of the other four genes from the Beineke model included in the larger model? If so, how does the model perform when these genes and correlated genes are removed? It would be useful to know how critical these five genes are to the prediction of smoking status in general, and how significant the additional genes are.

7) Why did the authors not consider including exon/isoform annotations from additional curated sources such as GENCODE as others have done? The list of exons and isoforms in ENSEMBL is known to be incomplete (e.g. as mentioned in https://pubmed.ncbi.nlm.nih.gov/28968689/).
8) Although the reasoning for the Isoform Mapping Layer is obvious, the motivation for the Feature Selection Layer could be made clearer. At present, it is unclear how the authors conceived of the FSL, or why they think it improved model performance.
9) The model is interesting and there are relevant methodological innovations in this paper, but the subsequent analysis of the trained model and results could be improved. Importantly, this paper is missing an in-depth analysis that would hint at or suggest novel biology or new avenues for future investigation. Thus, its biological relevance could be strengthened. Given the authors' expertise in the biological aspects of this paper, I think this could be easily remedied. However, its current focus is mostly on machine learning. For instance, it would be interesting to see a more in-depth investigation of the trained models using one of the many interpretation algorithms (e.g. github.com/marcoancona/DeepExplain , github.com/slundberg/shap , and others) to identify which exons, genes, or isoforms proved most important to the algorithm's classification decisions. This might help explain the otherwise-opaque decisions of their neural network model, and improve readers' trust and confidence in the algorithm described. Without a more in-depth exploration of the model itself, this work comes across more as a computational improvement than one focused on both computational and biological aspects.
10) It is presently unclear how long each model configuration was trained for. The authors only mention measuring cross-validated performance. However, it is generally common to train a model for several epochs on the available data before the optimal weights are identified. Did they use any early stopping? The authors should also mention in the methods section what GPUs they used to train their models, and how long the training process took.
11) The figures currently appear to be rather low resolution. It would greatly improve their readability if the authors uploaded higher resolution versions of them.

Minor Points:

1) In the abstract, model performance is referred to in terms of the AUC, but it is not immediately obvious what curve they are referring to. It is only when one finally sees Figure 1 that AUC is revealed to be the

area under the receiver operating characteristic. Since there are other relevant metrics in this domain (e.g. AUPRC), the authors should make it clear that they are referring to the AUROC.

2) The authors should write out "RNA integrity number (RIN)" on first usage of "RIN".
3) The authors should indicate what parameter settings were used with the STAR aligner.
4) In the layer-by-layer architecture selection approach, did the authors retain the weights for early layers when they added subsequent layers, or were the weights for these layers randomly reinitialized?
5) The authors mention trying a fully connected exon-to-isoform mapping layer, and that it did not attain a suitable loss despite the possible configurations for this network subsuming the set of those for the non-fully-connected approach that did work. Why did they not try a gene-level mapping layer as well, where exons are connected to the genes they are associated with?
6) I see no serious issues with the author's network architecture selection method, but I do wonder if it could have been improved. Why was a layer-by-layer approach used for model selection rather than a simultaneous search over a joined space of all layer configurations and counts (i.e. via neural architecture search, or sequential model-based optimization, etc.)? There is extensive work on the latter, and neural architecture search methods have repeatedly outperformed manually-designed networks and more informal search procedures (e.g. see https://arxiv.org/abs/1908.00709 for more details). This is more of a question of interest than a criticism of their work, so the authors need not address this if they are short on time.
