# Supplementary material for: Improved prediction of smoking status via isoform-aware RNA-seq deep learning models
Source: PLoS Comput Biol. 2021 Oct 11;17(10):e1009433. doi: 10.1371/journal.pcbi.1009433 (PMC8530282; doi:10.1371/journal.pcbi.1009433)
Supplement: Attachment — Submitted filename: response_letter.pdf [file pcbi.1009433.s008.pdf]

April 26, 2021

Dr. Florian Markowetz
Cancer Research UK Cambridge Institute
Li Ka Shing Centre
Robinson Way
Cambridge, CB2 0RE, UK

Dr. Donna K. Slonim
Department of Computer Science
Tufts University
Medford, MA 02155

Dear Dr. Markowetz and Dr. Slonim,

Thank you for the invitation to respond to reviewer critiques for our article titled “Improved prediction of smoking status via isoform-aware RNA-seq deep learning models” to be considered for publication as an original research article. We appreciate the thoughtful critiques of the reviewers, and we have conducted new analyses and made changes to the text of the article as described in detail below. These changes have strengthened the manuscript, and we hope that you will find it suitable for publication in PLoS Computational Biology.

In this article, using blood RNA-seq data from 2,557 subjects in the COPDGene Study, we demonstrate for the first time how isoform variability acts as an important source of latent information in RNA-seq data that improves the accuracy of prediction models for current smoking status. This manuscript makes a strong case for encoding biological information into a deep learning model, and it provides comprehensive experimental results on datasets of large sample size.

Dr. Peter J. Castaldi is the corresponding author for this manuscript. His telephone number is 617-636-7359, and his email is peter.castaldi@channing.harvard.edu. The mailing address is: Channing Division of Network Medicine/Brigham and Women’s Hospital/181 Longwood Avenue/Boston, MA 02115.

We appreciate your consideration of this manuscript.
Sincerely,

Peter J. Castaldi, MD, MSc
Assistant Professor of Medicine
Channing Division of Network Medicine
Brigham and Women’s Hospital
Harvard Medical School

Reviewer 1
<u>Major points:</u>
1) The current introduction does little to frame the work's methodological contributions and innovations with respect to the existing literature on deep learning applications for computational biology. This is unfortunate due to the apparent novelty of the isoform map layer and other important contributions. Most of the introduction rehashes the authors' previous contributions to the field of transcriptomics analysis, or references specific biological mechanisms (e.g. T-cell activation) relevant to their data that are mentioned nowhere else in the paper. It would be appropriate to have at least one reference and sentence mentioning deep learning for computational biology in specific (e.g. one of the many reviews out there, such as dx.doi.org/10.1098/rsif.2017.0387 or another similar review). To a lesser extent, it may also be worth noting the several previous applications of deep learning to splicing and isoforms (e.g. most notably dx.doi.org/10.1126/science.1254806 and www.nature.com/articles/s41592-019-0351-9), although the focuses of such works have been different from this one's.

Author response:
We agree that more thorough discussion of relevant background literature would benefit the introduction, and we have accordingly added the text below.

Introduction (lines 18-36)
High throughput measurements of gene expression in biological samples have been shown to capture information relevant to complex biological processes such as cell cycle [PMID: 10963673], stress response [PMID: 9843981], and medical disease states [PMID: 22447773]. Gene expression-based multigene predictive models have achieved a level of performance that has resulted in their regular use in medical decision making, most notably in early stage breast cancer [PMID: 15591335], but this level of precision has not yet been attained in many other areas of clinical practice. More recent research has applied neural networks to gene expression data in biomedical domains [PMID: 29618526], achieving superior performance relative to other machine learning methods in some cases [PMID: 31825821], though this is not a universal finding [PMID: 32197580]. While gene expression microarrays were first used for genomewide transcriptomics profiling, massively parallel high-throughput RNA sequencing (RNA-seq) is now the standard, and one of the benefits of RNA-seq is that it can directly measure exon expression and detect junctional reads (i.e. RNA-seq reads spanning exons) which allows for estimation of transcript isoforms. It has been shown that the additional information that RNA-seq provides on alternative splicing allows for more sensitive detection of transcriptomic differences between cancer subtypes, but this information did not necessarily lead to improved prediction of clinical outcomes [PMID: 26109056], suggesting that there may be latent information in RNA-seq data related to splicing that may require novel modeling approaches to better utilize this information.

2) Although the transcriptomic data for the manuscript has been uploaded to GEO, I could not find the list of specific gene/exon/isoform covariates that were used in the models. While the list of 1,270 genes is readily available in Huan et al., the others are not.

The authors do give the version of ENSEMBL that they used, and some vague instructions on how to derive and filter the isoforms and exons. However, it would behoove the authors to also include the exon and isoform definitions themselves as supplementary data. At present the reproducibility of their entire paper hinges entirely on this point, as well as on Biomart's continued support and availability of old releases. Nevertheless, even if one acquires the correct GTF and performs the procedures in the methods, there is no way to verify that the resultant sets and definitions exactly match the ones used in the paper. Ideally, the authors would also include the code used to derive the exon definitions and reproduce their paper and archive it publicly (e.g. on Zenodo or elsewhere).

Author response:
We agree that it is important to include the exon and isoform definitions, as well as the code to derive the exon definitions. We have uploaded these files to https://github.com/KingSpencer/COPD-IsoformMap accordingly. Specifically:

- The code and instructions to derive the exon and isoform definitions
  - https://github.com/KingSpencer/COPD-IsoformMap/blob/main/deeplearning_geneAnnotation.html (Please download this file and view using your browser)
- The code to reproduce our paper
  - https://github.com/KingSpencer/COPD-IsoformMap
- Files containing the list of genes, isoforms, exons
  - https://github.com/KingSpencer/COPD-IsoformMap/tree/main/mapping_data
- Files containing gene-exon, isoform-exon mapping
  - Gene-exon:https://drive.google.com/file/d/11qG9uAmLuXgL-x3HKR8jRXfUoPXLISkF/view?usp=sharing
  - Isoform-exon:https://drive.google.com/file/d/1jzu9uXVIheKc69kqCp08Kx3AEXdOAWDd/view?usp=sharing

Moreover, we have added essential information about the software artifacts in our manuscript:

Materials and methods (lines 129-131)
All network definitions, network weights and code, as well as additional files required for reproducing our experiment results are available at https://github.com/KingSpencer/COPD-IsoformMap

3) I could not find a full description of the network architecture used for each set of covariates. This is critical to understanding the paper, and it is incomplete without it. The authors have also left out the learned weights for their neural network model, as well as the code used for their model. These software artifacts are essential to reproducing and understanding this paper, since subtle implementation differences can lead to drastically different outcomes.

Author response:
To address this concern, we have added the full description of the network architectures added in the manuscript in Table 4.

Table 4. Corresponding network architectures.

	Architecture
Exon Base	Input-256-128-64-Output
Exon, GML-GTF	Input-GML-128-64-32-Output
Exon, GML-GTF, FSL	Input-GML-FSL-128-64-32-Output
Exon, IML-GTF	Input-IML-256-128-64-Output
Exon, IML-GTF, FSL	Input-IML-FSL-256-128-64-Output

IML-GTF: Isoform Map Layer containing information from GTF file. GML-GTF: Gene Map Layer containing information from Ensembl GTF file. FSL: Feature Selection Layer. Each number represents a fully connected layer with that number of nodes.

Moreover, as we have responded to the reviewer's major comment 2), we have created a GitHub repository containing all essential software artifacts:
https://github.com/KingSpencer/COPD-IsoformMap

To be specific, the network definitions are in:
https://github.com/KingSpencer/COPD-IsoformMap/tree/main/utils
Due to the file size, we can only provide a link on github containing the network weights. For your information, the actual link is provided here:
https://drive.google.com/file/d/1XHQXM9cA1IX2jZVp5Hp6LzOCZEihqi9y/view?usp=sharing

4) The discussion of cotinine and the model's applicability has a few issues. For instance, the authors mention that the model could be used in scenarios where transcriptomic data are available, but cotinine measurements are not. The authors also state that their model performs worse than cotinine measurements for classifying smoking status. However, this is merely assumed based on cotinine's performance as a predictor on entirely different datasets. It would be more accurate for the authors to instead state that cotinine measurements are known to be a strong predictor of smoking status, but it is unknown how their model will compare to them. Clearly, this is not ideal. Thus, if there exists a dataset of paired cotinine and RNA-seq expression data, then evaluation on said dataset with the author's model seems like a needed addition.

Author response:
We appreciate this excellent suggestion from the reviewer. Plasma metabolite data is available for a subset of subjects from COPDGene. We obtained these data and compared the discriminative performance of cotinine and the exon model using the isoform map layer with feature selection in 106 individuals from the test set. Interestingly, the exon model clearly

outperforms plasma cotinine in these data, which we have described in updated text in the Materials and methods, Results and Discussion sections.

Materials and methods (lines 181-186)
Cotinine measurements were obtained from plasma through metabolomic profiling using the Metabolon Global Metabolomics Platform (Durham, NC, USA) . The data were further normalized to remove batch effects. Samples with undetectable cotinine levels were assigned a value of zero. COPDGene metabolomic data is available at the NIH Common Fund's National Metabolomics Data Repository (NMDR) website, the Metabolomics Workbench, https://www.metabolomicsworkbench.org (Study ID ST001443) .

Results (lines 242-250)
Cotinine is a metabolite of nicotine and the most commonly used biomarker for current smoking status. Out of 513 subjects in the test dataset, 106 had plasma metabolite measurements available for analysis in which we could compare the performance of predicted smoking status from the exon IML-GTF FSL model to that of plasma cotinine values. Interestingly, in these data the predictions from the exon-level model significantly outperformed plasma cotinine (DeLong p-value = 0.01, Figure 5), and the distribution of cotinine levels and exon predicted values in current and former smokers is shown in Supplemental Figures S1 and S2.

Discussion (lines 317-320)
In our dataset, predictions from exon expression using an isoform mapping layer achieved a sensitivity of 83% with a specificity of 89%, and when compared directly to plasma cotinine levels in a subset of subjects from COPDGene, our exon expression predictive model significantly outperformed cotinine.

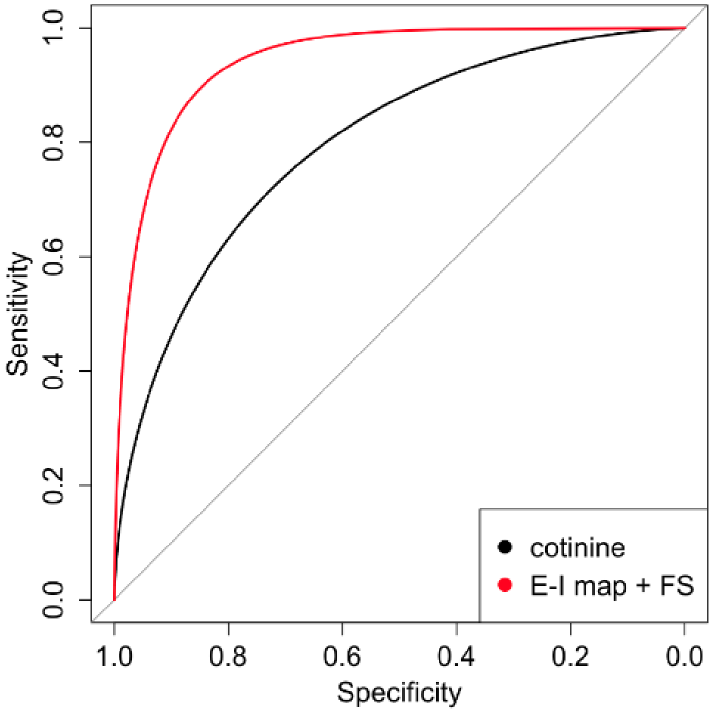

Fig 5. ROC curves in test data for the serum cotinine (black) and the exon model including the (Exon-to-)Isoform Map Layer and Feature Selection Layer (red) which has significantly better performance (Delong test p=0.01).

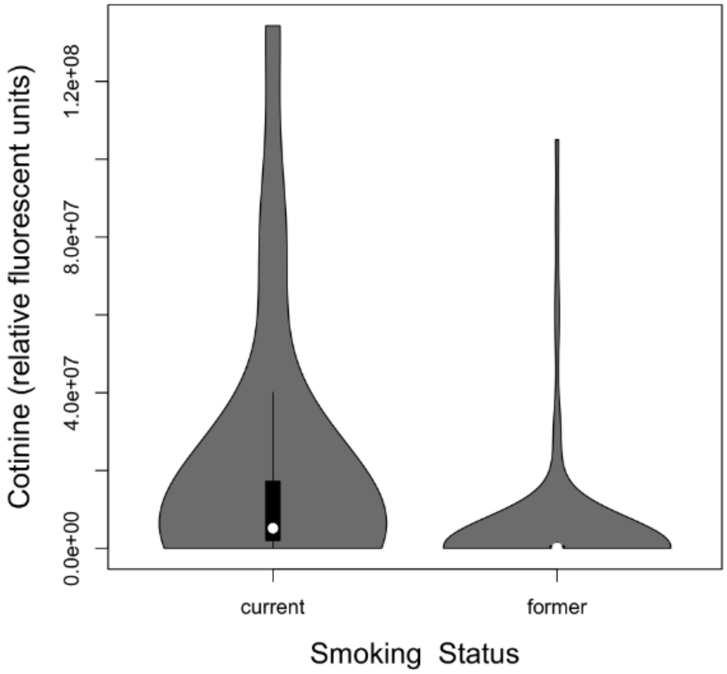

Fig S1. The distribution of cotinine levels in current and former smokers (N=21 and 85, respectively).

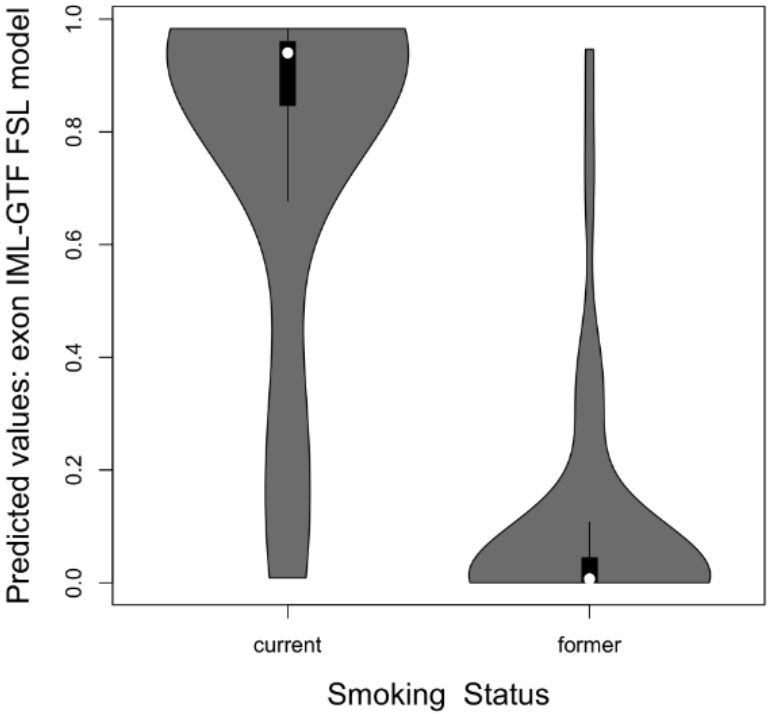

Fig S2. The distribution of exon predicted values in current and former smokers (N=21 and 85, respectively).

5) It appears that they normalized their training/validation/testing data using the trimmed mean of M values (TMM) implementation in the edgeR library. However, since the authors have not included their source code, it is not immediately obvious which samples were chosen as reference samples for the normalization step. This turns out to be critical. If the reference sample was included in the validation or testing data, then it represents a leakage of test set information and could lead to inflated test performance estimates. It is entirely possible that this is not the case however. Hopefully, the authors can clear up this confusion. In general, an evaluation on additional RNA-seq data from another cohort would more convincingly demonstrate the model's ability to generalize beyond the COPDGene cohort and RNA-seq batches.

Author response:
To address this issue we re-normalized our data using upper-quartile normalization that computes self-contained normalization factors without relying on a single reference sample, and we repeated all of our analyses in this re-normalized data. As can be seen in Supplemental Tables S1 and S2, our results are consistent using both TMM and upper-quartile normalization, indicating that the performance of our models is robust to the choice of normalization methods and is not subject to leakage of information through the normalization procedure.

We recognize the importance of replication in another cohort, but unfortunately we could not identify a suitable independent replication cohort with available smoking data and an RNA-seq library prep protocol that matches the one used in this COPDGene dataset (i.e. PaxGene RNA extraction and total RNA preparation with globin reduction, not the more standard polyA-selection). The difference between total RNA preparation (i.e. ribosomal reduction only) and poly-A selection has previously been shown to have a significant impact on the identified RNA species and expression profiles [PMIDs 20688152].

We feel that our stringent test and training design and the large size of our test data are a strength of this study that provide a good level of confidence in the validity of these results, but to clarify the critical importance of future work to develop models that may be translatable to clinical practice we added a sentence to the Discussion emphasizing the importance of independent replication. We hope to address this issue in more depth in future work when RNA-seq data from other large comparable studies, such as SPIROMICS, are publicly available.

Discussion (lines 334-336)
While our models performed well in held-out test data, further validation and replication of these results in other cohorts with similar RNA isolation and sequencing protocols is necessary prior to clinical translation.

Table S1. Predictive performance of modified Beineke models using gene, isoform and exon-level expression data, with *MUC1* included and upper quantile normalized.

	Val - Accuracy	Val - AUC	Test - Accuracy	Test - AUC
Gene	0.673	0.687	0.698	0.752
Isoform	0.781	0.807	0.803	0.794
Exon	0.808	0.844	0.808	0.869
Exon, IML-GTF	**0.828**	**0.874**	**0.840**	**0.871**
Exon, IML-GTF, FSL	0.818	0.863	0.813	0.869

Val: validation data. AUC: area under the receiver operating characteristic. IML-GTF: Isoform Map Layer containing information from Ensembl GTF file. FSL: Feature Selection Layer. Best results are shown in bold.

Table S2. Predictive performance of various deep learning models using exon-level data processed with upper-quartile normalization.

	Val - Accuracy	Val - AUC	Test - Accuracy	Test - AUC
Exon Base	0.832	0.895	0.854	0.909
Exon, IML-GTF	0.847	0.916	0.856	0.918
Exon, IML-GTF, FSL	**0.858**	**0.916**	**0.858**	**0.936**

Val: validation data. AUC: area under the receiver operating characteristic. IML-GTF: Isoform Map Layer containing information from Ensembl GTF file. FSL: Feature Selection Layer. Best results are shown in bold.

6) Does inclusion of *MUC1* have a significant effect on the model performance? I realize that it has a low abundance, but that does not necessarily mean that it is irrelevant or would not influence the model performance in a significant way. At present, since *MUC1* was not included in their Beineke-based model, it does not seem like there has been a proper evaluation of the original Beineke model. Along those lines, are any or all of the other four genes from the Beineke model included in the larger model? If so, how does the model perform when these genes and correlated genes are removed? It would be useful to know how critical these five genes are to the prediction of smoking status in general, and how significant the additional genes are.

Author response:

a. To address the first concern, we include *MUC1* in our analysis and repeat the same analysis on the Beineke-based models, and we have added all results in Supplemental Table S1. For your convenience, the original Table 2 containing results without *MUC1* is also copied here.

We have also added text descriptions in the Supplementary material accordingly:

Supplementary material (line 10-12)

Despite the low level expression of *MUC1*, we added *MUC1* to the set of genes, and the corresponding isoform and exon features for completeness. The results in Supplemental Table S1 are close to the results shown in our main text for all exon based models.

Table 2. Predictive performance of modified Beineke models using gene, isoform and exon-level expression data.

	Val - Accuracy	Val - AUC	Test - Accuracy	Test - AUC
Gene	0.698	0.758	0.743	0.780
Isoform	0.757	0.828	0.774	0.828
Exon	0.801	0.859	0.808	0.869
Exon, IML-GTF	**0.828**	0.876	0.825	0.870
Exon, IML-GTF, FSL	**0.828**	**0.889**	**0.838**	**0.875**

Val: validation data. AUC: area under the curve. IML-GTF: Isoform Map Layer containing information from Ensembl GTF file. FSL: Feature Selection Layer. Best results are shown in bold.

Table S1. Predictive performance of modified Beineke models using gene, isoform and exon-level expression data, with *MUC1* included and upper-quartile normalized.

	Val - Accuracy	Val - AUC	Test - Accuracy	Test - AUC
Gene	0.673	0.687	0.698	0.752
Isoform	0.781	0.807	0.803	0.794
Exon	0.808	0.844	0.808	0.869
Exon, IML-GTF	**0.828**	**0.874**	**0.840**	**0.871**
Exon, IML-GTF, FSL	0.818	0.863	0.813	0.869

Val: validation data. AUC: area under the receiver operating characteristic. IML-GTF: Isoform Map Layer containing information from Ensembl GTF file. FSL: Feature Selection Layer. Best results are shown in bold.

b. All the four genes from the Beineke model are included in the larger model. To address the second concern, we remove the 4 other genes as well as correlated genes (correlation coefficient >= 0.4) in our original dataset (TMM normalized) with a larger feature set. The result is shown in Supplemental Table S3. We have also added text descriptions accordingly:

Supplementary material (line 15-22)
To assess the importance of the genes used in the Beineke model, we remove these genes together with their correlated genes (with correlation coefficient >= 0.4) from the larger feature set of 1079 genes, resulting in 1020 uncorrelated genes. We use the base deep learning model for gene input. The final result is shown in Supplemental Table S3. Comparing the test AUC (0.900 versus 0.856), we can see that removing these genes and their correlated genes indeed results in a decrease in performance. However, based on the information contained within the remaining uncorrelated genes, our model is able to give reasonable predictive results.

Table S3. Predictive performance of deep learning models using all 1079 genes, and 1020 genes with genes used in the Beineke model and their correlated genes (correlation coefficient ≥ 0.4) removed.

	Val - Accuracy	Val - AUC	Test - Accuracy	Test - AUC
1079 Gene, Base	**0.828**	**0.900**	**0.830**	**0.900**
1020 Gene Uncorrelated, Base	0.788	0.825	0.782	0.856

Val: validation data. AUC: area under the receiver operating characteristic. Base: the base architecture for gene input, Input-128-64-32-Output. Best results are shown in bold.

7) Why did the authors not consider including exon/isoform annotations from additional curated sources such as GENCODE as others have done? The list of exons and isoforms in ENSEMBL is known to be incomplete (e.g. as mentioned in https://pubmed.ncbi.nlm.nih.gov/28968689/).

Author response:
Our understanding is that the correspondence between Ensembl and GENCODE has been established since GENCODE version 3c (equivalent to Ensembl 56) [PMID: 25765860]. Per the Ensembl FAQs (https://useast.ensembl.org/Help/Faq?id=303), the default human and mouse gene sets in the Ensembl browser are provided to GENCODE as the current version. And per the GENCODE FAQs (https://www.gencodegenes.org/pages/faq.html), there is now essentially no difference between the GENCODE and ENSEMBL GTFs. To verify this critical point we compared the Ensembl GTF (release 94) used in our analysis to the corresponding GENCODE GTF (release 29), and we confirmed the correspondence between these two curation resources (see https://github.com/KingSpencer/COPD-IsoformMap/blob/main/deeplearning_geneAnnotation.html).

8) Although the reasoning for the Isoform Mapping Layer is obvious, the motivation for the Feature Selection Layer could be made clearer. At present, it is unclear how the authors conceived of the FSL, or why they think it improved model performance.

Author response:
Thanks for this constructive suggestion. We have made the motivation for the FSL clearer in our manuscript:

Materials and methods: (line 158-163)
To enhance interpretability, we included in some models a Feature Selection Layer (FSL) that associates every input feature with a non-negative learnable weight using an L1 constraint and outputs a reweighted feature vector of the same size as the input feature vector. Since the weights are non-negative, they can be considered to represent each feature's importance with respect to smoking status prediction, and the L1 constraint is meant to improve the generalizability of the model.

9) The model is interesting and there are relevant methodological innovations in this paper, but the subsequent analysis of the trained model and results could be improved. Importantly, this paper is missing an in-depth analysis that would hint at or suggest novel biology or new avenues for future investigation. Thus, its biological relevance could be strengthened. Given the authors' expertise in the biological aspects of this paper, I think this could be easily remedied. However, its current focus is mostly on machine learning. For instance, it would be interesting to see a more in-depth investigation of the trained models using one of the many interpretation algorithms (e.g. github.com/marcoancona/DeepExplain , github.com/slundberg/shap , and others) to identify which exons, genes, or isoforms proved most important to the algorithm's classification decisions. This might help explain the otherwise-opaque decisions of their neural network model, and improve readers' trust and confidence in the algorithm described. Without a more in-depth exploration of the model itself, this work comes across more as a computational improvement than one focused on both computational and biological aspects.

Author response:
We agree that model interpretation is vital and could add more biological relevance, and we appreciate the authors suggestion to consider the DeepExplain framework. To strengthen the paper, we used DeepExplain with saliency maps to generate feature importance scores for 19,027 exons. Moreover, we added a section called *Model interpretation* in the manuscript to show our analysis, with new figures and tables. The corresponding modifications in our manuscript are as follows:

Materials and methods: (line 175-179)
Gene set functional enrichment analysis was performed for genes ranked in the top 20% of feature importance scores generated by the DeepExplain [20] framework with saliency maps [21]. We used the TopGO package [22] to compute the gene set enrichment p-values for Gene Ontology pathways using the 'weight01' algorithm with Fisher's exact test statistic.

Results: (line 251-260)
Model interpretation:
We explored the interpretation of our best performing model, (the Exon, IML-GTF, FSL model), and we generated corresponding feature importance scores for each exon using the DeepExplain [20] framework with saliency maps [21]. 48.5% of exons had non-zero scores, and the distribution of the non-zero scores was bimodal (Supplemental Figure S3). We selected the exons in the top 20% of saliency scores for gene pathway enrichment analysis using the TopGO method [22] with the 1,079 analyzed genes as the background for comparison, and 43 Gene Ontology pathways had nominally significant p-values with the most enriched pathways related to GTPase activity and protein ubiquitination/degradation. The top 10 pathways are shown in Table 5.

Table 5. Top 10 enriched GO pathways.

Go.ID	Term	Annotated	Significant	Expected	p-value
GO:0032092	positive regulation of protein binding	9	6	1.87	0.0036
GO:0043547	positive regulation of GTPase activity	24	11	4.99	0.005
GO:0031397	negative regulation of protein ubiquitination	9	5	1.87	0.0076
GO:0006892	post-Golgi vesicle-mediated transport	5	4	1.04	0.0076
GO:0006998	nuclear envelope organization	5	4	1.04	0.0076
GO:0015696	ammonium transport	5	4	1.04	0.0076
GO:0032722	positive regulation of chemokine production	5	4	1.04	0.0076
GO:0010950	positive regulation of endopeptidase activity	17	8	3.53	0.0086
GO:0032885	regulation of polysaccharide biosynthetic process	3	3	0.62	0.0089
GO:0048199	vesicle targeting, to, from or within Golgi	3	3	0.62	0.0089

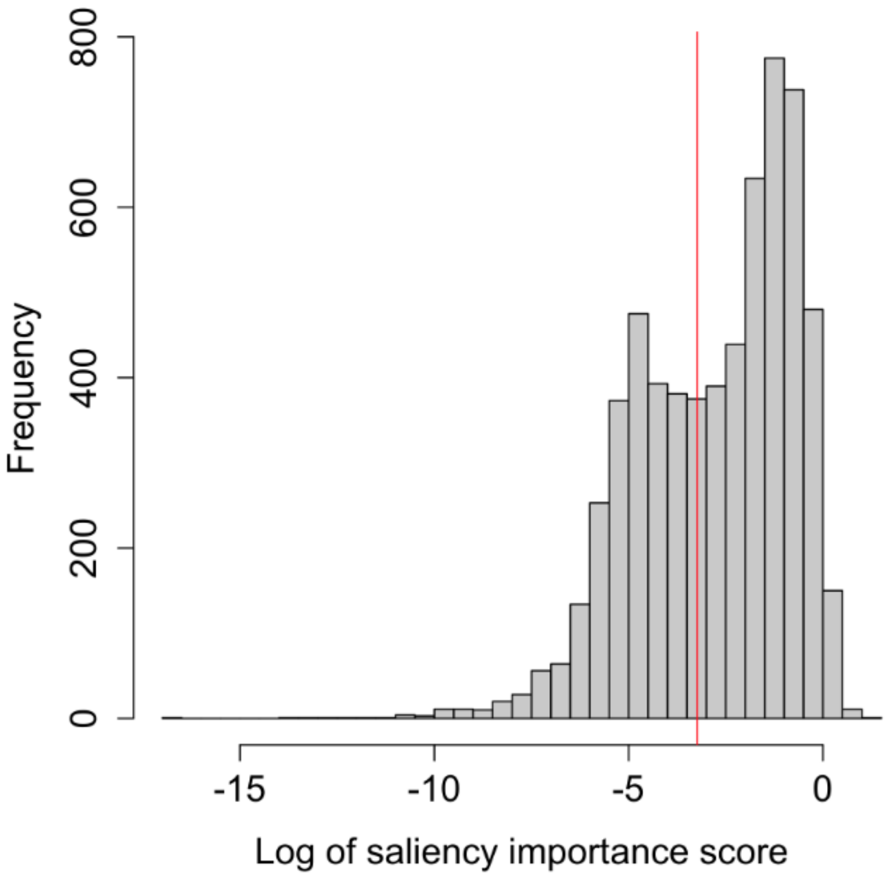

Fig S3. The distribution of log of feature importance scores for each exon using the DeepExplain [20] framework with saliency maps [21] on the trained Exon, IML-GTF, FSL model. 48.5% of exons had non-zero scores, and the distribution of the 243 non-zero scores was bimodal. The red line in the figure indicates the top 20% of exons.

10) It is presently unclear how long each model configuration was trained for. The authors only mention measuring cross-validated performance. However, it is generally

common to train a model for several epochs on the available data before the optimal weights are identified. Did they use any early stopping? The authors should also mention in the methods section what GPUs they used to train their models, and how long the training process took.

Author response:
Thanks for pointing out this issue. We have added corresponding training details in the manuscript:

Materials and methods: (line 125-126)
We employ the early stopping strategy to get the best performing model on the validation set.

Materials and methods: (line 132-135)
All experiments are conducted on a single NVIDIA GTX 1080Ti GPU. The training process related to Beineke models ranges from 13 seconds to 17 seconds for 40 epochs. The training process related to a larger feature set ranges from 58 seconds (the base model) to 252 seconds (model with IML and FSL) for 40 epochs.

11) The figures currently appear to be rather low resolution. It would greatly improve their readability if the authors uploaded higher resolution versions of them.

Author response:
We have generated figures of higher resolution, please see the newly submitted figure files.

Minor Points:
1) In the abstract, model performance is referred to in terms of the AUC, but it is not immediately obvious what curve they are referring to. It is only when one finally sees Figure 1 that AUC is revealed to be the area under the receiver operating characteristic. Since there are other relevant metrics in this domain (e.g. AUPRC), the authors should make it clear that they are referring to the AUROC.

Author response:
We have made it clear in the abstract that we are referring to AUROC (Abstract, line 11-12).

2) The authors should write out “RNA integrity number (RIN)” on first usage of “RIN”.
We have made the corresponding change in our manuscript (Materials and methods, line 67).

3) The authors should indicate what parameter settings were used with the STAR aligner.

Author response:

We have provided STAR parameter settings in our manuscript:

Materials and methods: (line 71-77)
We used these arguments for the first pass: STAR --runThreadN 8 --outSAMunmapped Within --outSAMstrandField intronMotif --outSJfilterReads Unique --outSJfilterCountUniqueMin 100 1 1 1. For the second pass, we provided splice junctions from the first pass with these additional arguments: --outSAMtype BAM SortedByCoordinate --limitSjdbInsertNsj 10000000 --chimSegmentMin 10 --sjdbFileChrStartEnd SJ.out.tab.

4) In the layer-by-layer architecture selection approach, did the authors retain the weights for early layers when they added subsequent layers, or were the weights for these layers randomly reinitialized?
Author response:
We made this confusion clear in our manuscript:

Materials and methods: (line 142-144)
Note that we reinitialize the weights of the previous layers when searching for the current layer.

5) The authors mention trying a fully connected exon-to-isoform mapping layer, and that it did not attain a suitable loss despite the possible configurations for this network subsuming the set of those for the non-fully-connected approach that did work. Why did they not try a gene-level mapping layer as well, where exons are connected to the genes they are associated with?

Author response:
We added additional experiments with a gene-level mapping layer, called Gene Map Layer (GML) and showed the corresponding result as additional rows in Table 2 and 3 respectively. We can see that no improvement from the GML could be observed compared with the Exon Base model.

We also updated the corresponding text in our manuscript:

Materials and methods: (line 155-157)
Analogously, we can devise an isoform-to-gene mapping layer, where exons are connected to the genes they are associated with, in the same way. We call it Gene Map Layer (GML).

Results: (line 239-241)
However, there was no improvement from the Gene Map Layer or from providing exon and isoform quantifications directly to the Elastic Net model without any exon-to-isoform relationship information.

Table 2. Predictive performance of modified Beineke models using gene, isoform and exon-level expression data.

	Val - Accuracy	Val - AUC	Test - Accuracy	Test - AUC
Gene	0.698	0.758	0.743	0.780
Isoform	0.757	0.828	0.774	0.828
Exon	0.801	0.859	0.808	0.869
Exon, GML-GTF	0.771	0.807	0.789	0.811
Exon, GML-GTF, FSL	0.776	0.805	0.741	0.796
Exon, IML-GTF	**0.828**	0.876	0.825	0.870
Exon, IML-GTF, FSL	**0.828**	**0.889**	**0.838**	**0.875**

Val: validation data. AUC: area under the receiver operating characteristic. IML-GTF: Isoform Map Layer containing information from Ensembl GTF file. GML-GTF: Gene Map Layer containing information from Ensembl GTF file. FSL: Feature Selection Layer. Best results are shown in bold.

Table 3. Predictive performance of various models using exon-level data, including elastic net for comparison.

	Val - Accuracy	Val - AUC	Test - Accuracy	Test - AUC
Exon, Elastic Net	0.821	0.861	0.774	0.903
Exon + Iso, Elastic Net	0.808	0.884	0.766	0.884
Exon Base	0.813	0.886	0.842	0.913
Exon, GML-GTF	0.833	0.899	0.842	0.913
Exon, GML-GTF, FSL	0.850	0.903	0.838	0.919
Exon, IML-GTF	0.843	0.905	0.854	0.924
Exon, IML-GTF, FSL	**0.860**	**0.916**	**0.869**	**0.935**

Val: validation data. AUC: area under the receiver operating characteristic. Exon + Iso: Concatenation of exon and isoform data. IML-GTF: Isoform Map Layer containing information from GTF file. GML-GTF: Gene Map Layer containing information from Ensembl GTF file. FSL: Feature Selection Layer. Best results are shown in bold.

6) I see no serious issues with the author's network architecture selection method, but I do wonder if it could have been improved. Why was a layer-by-layer approach used for model selection rather than a simultaneous search over a joined space of all layer configurations and counts (i.e. via neural architecture search, or sequential model-based optimization, etc.)? There is extensive work on the latter, and neural architecture search methods have repeatedly outperformed manually-designed networks and more informal search procedures (e.g. see https://arxiv.org/abs/1908.00709 for more details). This is more of a question of interest than a criticism of their work, so the authors need not address this if they are short on time.

Author response:

This is an excellent point, however, neural architecture search is computationally expensive and out of the scope of the research objective of this work. It will be interesting to add this analysis in our future work.

Reviewer 2

1) The model, particularly the deep net with the larger set of features, could actually be picking up on features that are directly associated with covariates (e.g., age, sex, BMI), which could then have associations with smoking status. In other words, the model may be predicting these covariates, not smoking status directly, which is dangerous when applying the model to other populations. The authors need to show that the model is truly learning expression signatures that are directly related to smoking, and not these covariates.

Author response:

We appreciate the reviewer's point, which is that our prediction models might be predicting key demographic covariates rather than smoking status directly, potentially jeopardizing the generalization to other populations with different demographic characteristics. To evaluate this, we constructed gene, exon, and isoform datasets in which the effects of age, sex, and BMI had been regressed out of the count data. In new experiments training models on these data, we observe a decrease in the predictive performance of these models (AUC for the exon IML-FS model decreased from 0.94 to 0.91), but this difference was not statistically significant (DeLong p-value = 0.09). This analysis suggests our models' performance is not dependent on these demographic variables. These results have been included as Supplemental Table S5, and we added a section to the results which is included below.

Results (lines 261-270)

To test whether the high performance of the RNA-seq models might be tied to specific demographic characteristics of the COPDGene study population such as age, sex, or body mass index, we fitted a linear model of the expression data using these demographic covariates together with smoking status as explanatory variables, and generated an adjusted version of the expression data by removing the effects of these covariates while retaining the main effect from smoking. We observed in the adjusted expression data a small but non-significant decrease in predictive performance for the exon-level model (AUC 0.91 versus 0.94, DeLong p-value = 0.09). These results are reported in Supplemental Table S5.

Table S5. Predictive performance of various deep learning models using the full set of exon-level data, with covariates signals removed from the data.

	Val - Accuracy	Val - AUC	Test - Accuracy	Test - AUC
Exon Base	0.830	0.884	0.832	0.901
Exon, IML-GTF	**0.867**	0.921	0.834	0.900
Exon, IML-GTF, FSL	0.867	**0.933**	**0.846**	**0.905**

Val: validation data. AUC: area under the receiver operating characteristic. IML-GTF: Isoform Map Layer containing information from Ensembl GTF file. FSL: Feature Selection Layer. Covariates removed: age, sex, body-mass index, and batch. Best results are shown in bold.

2) Related to 1, the authors do not characterized the features that the learned models are picking up on and what biology these might suggest with respect to smoking status. For a paper to PLoS CompBio, I would expect more characterization of the molecular biology.

Author response:
Thanks for pointing this out. We agree that characterizing the features learned by the models are important. And this point coincides with Reviewer 1's major point 9).
We have conducted gene set functional enrichment analysis for genes ranked by feature importance scores generated by DeepExplain framework with saliency maps, using the TopGO method. Please see the corresponding response to Reviewer 1's major point 9).

3) I'm not fully convinced that the deep net is outperforming a simple logistic regression model with similar features. In Table 3, I believe the "Elastic Net" model is a logistic regression model. If this model is also given isoform abundances (i.e., Exon + Isoform, Elastic Net), what is the performance? The IML layer is essentially giving isoform level information to the deep net, so for a fair comparison, this information should also be given to the logistic regression model. One really needs to show a big gain in performance with a deep net over a simpler model to justify its use, and I am not seeing such a difference here.

Author response:
We agree that a more fair comparison should be made by providing the logistic regression model with the isoform level information. In order to give the logistic regression model both exon and isoform information, we concatenate the exon and isoform feature into a single feature vector, and use that as the input of the logistic regression model. The result is shown as an additional row in Table 3. It seems that adding isoform features to the logistic regression model made the final test AUC worse, from 0.903 to 0.884. A possible explanation is that the logistic regression model is not able to take advantage of the additional exon-isoform mapping information while suffering from the extra collinearity introduced by isoform data.

We have also added additional text in our manuscript accordingly:

Results: (line 239-241)
However, there was no improvement from the Gene Map Layer or from providing exon and isoform quantifications directly to the Elastic Net model without any exon-to-isoform relationship information.

Table 3. Predictive performance of various models using exon-level data, including elastic net for comparison.

	Val - Accuracy	Val - AUC	Test - Accuracy	Test - AUC
Exon, Elastic Net	0.821	0.861	0.774	0.903
Exon + Iso, Elastic Net	0.808	0.884	0.766	0.884
Exon Base	0.813	0.886	0.842	0.913
Exon, GML-GTF	0.833	0.899	0.842	0.913
Exon, GML-GTF, FSL	0.850	0.903	0.838	0.919
Exon, IML-GTF	0.843	0.905	0.854	0.924
Exon, IML-GTF, FSL	**0.860**	**0.916**	**0.869**	**0.935**

Val: validation data. AUC: area under the receiver operating characteristic. Exon + Iso: Concatenation of exon and isoform data. IML-GTF: Isoform Map Layer containing information from GTF file. GML-GTF: Gene Map Layer containing information from Ensembl GTF file. FSL: Feature Selection Layer. Best results are shown in bold.

4) In the authors' previous study [3], they identified differentially expressed exons (while taking into account covariates!) associated with smoking status. Why are those not used in this study?

Author response:

Since we wanted to evaluate our models in test set data from COPDGene, we did not want to introduce bias by selecting genes that had been identified in previous analyses of these data. Thus, we chose to not make use of information from our previous differential expression analysis, and we instead limited our analysis to genes identified from the previous study by Huan et al., which was entirely independent of the data used to train and test our models. Please also see our response to comment 1) in which we describe the result of new experiments to better assess the impact of key covariates on the performance of these predictive models.
