# Supplementary material for: Improved prediction of smoking status via isoform-aware RNA-seq deep learning models
Source: PLoS Comput Biol. 2021 Oct 11;17(10):e1009433. doi: 10.1371/journal.pcbi.1009433 (PMC8530282; doi:10.1371/journal.pcbi.1009433)
Supplement: Attachment — Submitted filename: reviewer_comments_revision_1.pdf [file pcbi.1009433.s009.pdf]

General Opinion:

The authors have begun to address many of my concerns in their revised version of the manuscript, but many still remain.

Major Points:

1) The new additions to the introduction are an improvement and provide further context with which to evaluate this manuscript's contributions.
2) The authors have made an effort to improve the reproducibility and transparency of their manuscript by uploading the exon and isoform definitions to GitHub along with the code to produce said definitions. However, they should still upload this material to an open-access archive such as Zenodo or Dryad. Although it is rather accessible and widely-used, GitHub is not an open-access archive. Material uploaded to GitHub can be lost, edited, and made private. The authors could also include their network weights on such an archive, as they allow larger file uploads than GitHub. Finally, some of the code appears to be missing from the GitHub repository. For instance, the file "run.bash" makes reference to a file named "Training_keras.py", but this file is not included in the GitHub repository. This makes me concerned that the code they have uploaded is insufficient to reproduce their results.
3) In regards to documenting their model's architecture, the author's inclusion of Table 4 is a step in the right direction. However, the network descriptions should be included in a single place in the manuscript or supplemental information. After all, they are a critical component of the work. At present, some information (e.g. the use of ReLU nonlinearities) is presented in the "Model Training" subsection of the "Materials and Methods" section, while other information (e.g. the number of hidden units in a fully-connected layer) is only available in Table 4 and referenced in the "Improved prediction through Isoform Map and Feature Selection Layers" subsection of the "Results" section. A single unified visual description included in the supplement (i.e. something akin to Supplemental Figure S1 from [PMID:30661751]) and referenced in the "Methods" section would greatly improve the readability and coherency.
4) The author's comparison with cotinine-based classification of smoker status is an appropriate addition. However, it is not immediately clear what sort of model they used for the cotinine-based classification. I suspect it was a linear model, but this does not appear to be stated explicitly in the manuscript. Furthermore, it is worth noting that only 106 samples (i.e. ~21% of their test samples) included plasma metabolite profiling of cotinine levels, so the authors should mention this limitation in their discussion.
5) The reanalysis with upper quartile (UQ) normalization appears to be sound, and represents a significant effort by the authors to remedy the issues I raised regarding their normalization procedure. However, it does not sufficiently address my concerns regarding the trimmed mean of M values (TMM) normalization using a reference set containing elements from their test set. I remain adamant that they

should remove any results from any model trained on TMM-normalized data where the reference set includes examples from the test set. Although models trained with UQ-normalized data may produce similar performance metrics as those trained with TMM-normalized data (i.e. with test set information leakage), this is not the same as these models being the same. For instance, it is possible that the leakage of test set information into the TMM-based model will prevent it from generalizing well to new data. Given the sensitive issues and risks surrounding clinical classifiers, extra caution must be practiced here. Frankly, the authors should replace all TMM-based results (i.e. models, predictions, etc.) with those from UQ normalization, or simply re-apply TMM while explicitly using the set of training examples as TMM's reference set. Finally, in their discussion of the study's potential caveats, the authors should explicitly mention that their methods may not generalize to RNA-seq data generated with the more commonly used poly-A library selection method.

6) This is a useful analysis, but it is not directly comparable to their primary results (i.e. Table 2) because of the differences in normalization procedures. The authors should use a single normalization method throughout the manuscript, and then repeat this analysis. That being said, the removal of the Beineke model genes (which appears to have been done with a comparable normalization procedure) impacts model performance without totally abolishing it, which suggests that the authors gene set is a significant improvement over the five gene model and not just a rehashing of it.
7) My point was more concerned with the general incompleteness of commonly-used annotation sets of splice sites and isoforms rather than a comparison between ENSEMBL and GENCODE in specific. For instance, did the authors consider using novel splice sites from a database like Snaptron or even simply a large RNA-seq study like GTEx? Although such catalogs may be noisey, they may also include isoforms that are useful for this study. The authors need not repeat their analysis with such a set, but it could be worth mentioning as a potential future improvement.
8) This addition is an improvement and more clearly conveys the motivation behind the Feature Selection Layer (FSL). Could the authors explain in more detail why they expected the L1 constraint to improve generalizability or provide a citation supporting this notion?
9) The addition of the "Model Interpretation" section is a significant improvement in the manuscript. I would like more analysis in this direction however. For instance, how many of these genes are highly expressed in the lung or differentially expressed in smokers? Are there any isoforms or exons that appear important and are known to be specifically associated with smoking status or related phenotypes? In general, it would be nice to see if this model recapitulates known associations, and also whether it can be used to identify novel biology. Further analysis here would also greatly improve the demonstrated biological relevance of this work, and also provide and describe potential avenues for future biological research.

10) The authors have almost answered all of my questions about the training procedure. However, could they provide additional information regarding their early stopping strategy? In specific, what were their criteria for early stopping?
11) The authors have addressed my concerns regarding the readability of their uploaded figures.

Minor Points

All of the minor points have been sufficiently addressed.
