# Supplementary material for: Improved prediction of smoking status via isoform-aware RNA-seq deep learning models
Source: PLoS Comput Biol. 2021 Oct 11;17(10):e1009433. doi: 10.1371/journal.pcbi.1009433 (PMC8530282; doi:10.1371/journal.pcbi.1009433)
Supplement: Attachment — Submitted filename: Castaldi_response_2.pdf [file pcbi.1009433.s010.pdf]

August 8, 2021

Dr. Florian Markowetz
Cancer Research UK Cambridge Institute
Li Ka Shing Centre
Robinson Way
Cambridge, CB2 0RE, UK

Dr. Donna K. Slonim
Department of Computer Science Tufts University
Medford, MA 02155

Dear Dr. Markowetz and Dr. Slonim,

Thank you for the invitation to respond to reviewer critiques for our article titled “Improved prediction of smoking status via isoform-aware RNA-seq deep learning models” to be considered for publication as an original research article. We appreciate the thoughtful critiques of the reviewers, and in our second round of response to the reviewers we have conducted new analyses and made changes to the text of the article as described in detail below. These changes have strengthened the manuscript, and we hope that you will find it suitable for publication in PLoS Computational Biology.

In this article, using blood RNA-seq data from 2,557 subjects in the COPDGene Study, we demonstrate for the first time how isoform variability acts as an important source of latent information in RNA-seq data that improves the accuracy of prediction models for current smoking status. This manuscript makes a strong case for encoding biological information into a deep learning model, and it provides comprehensive experimental results on datasets of large sample size.

Dr. Peter J. Castaldi is the corresponding author for this manuscript. His telephone number is 617-636-7359, and his email is peter.castaldi@channing.harvard.edu. The mailing address is: Channing Division of Network Medicine/Brigham and Women’s Hospital/181 Longwood Avenue/Boston, MA 02115.

We appreciate your consideration of this manuscript.

Sincerely,

Peter J. Castaldi, MD, MSc
Associate Professor of Medicine
Channing Division of Network Medicine
Brigham and Women’s Hospital
Harvard Medical School

Reviewer 1
General Opinion:

The authors have begun to address many of my concerns in their revised version of the manuscript, but many still remain.

Major Points:

1) The new additions to the introduction are an improvement and provide further context with which to evaluate this manuscript's contributions.

2) The authors have made an effort to improve the reproducibility and transparency of their manuscript by uploading the exon and isoform definitions to GitHub along with the code to produce said definitions. However, they should still upload this material to an open-access archive such as Zenodo or Dryad. Although it is rather accessible and widely-used, GitHub is not an open-access archive. Material uploaded to GitHub can be lost, edited, and made private. The authors could also include their network weights on such an archive, as they allow larger file uploads than GitHub. Finally, some of the code appears to be missing from the GitHub repository. For instance, the file "run.bash" makes reference to a file named "Training_keras.py", but this file is not included in the GitHub repository. This makes me concerned that the code they have uploaded is insufficient to reproduce their results.

Author response:

Thanks for pointing out this issue. We have uploaded our code as well as key files to Zenodo, with DOI 10.5281/zenodo.5136729 and corresponding link https://doi.org/10.5281/zenodo.5136729.

Moreover, the "Training_keras.py" in "run.bash" turns out to be a typo, as we have renamed and reorganized the files. We have fixed this typo in our newest version.

We also updated the corresponding text in our manuscript:

Materials and methods (lines 132-134):

All network definitions, network weights and code, as well as additional files required for reproducing our experiment results are available at https://doi.org/10.5281/zenodo.5136729.

3) In regards to documenting their model's architecture, the author's inclusion of Table 4 is a step in the right direction. However, the network descriptions should be included in a single place in the manuscript or supplemental information. After all, they are a critical component of the work. At present, some information (e.g. the use of ReLU nonlinearities) is presented in the "Model Training" subsection of the "Materials and Methods" section, while other information (e.g. the number of hidden units in a fully-connected layer) is only available in Table 4 and referenced in the "Improved prediction through Isoform Map and Feature Selection Layers" subsection of the "Results" section. A single unified visual

description included in the supplement (i.e. something akin to Supplemental Figure S1 from [PMID:30661751]) and referenced in the "Methods" section would greatly improve the readability and coherency.

Author response:

Thanks for this constructive suggestion. We have removed the original Table 4 from the main manuscript, and added a visual summary of all deep learning models in Supplemental Figure S4 (see below). We have also updated the reference to this visual summary in the Methods section:

Materials and methods (lines 130-131):

A visualization of all network architectures can be found in the Supplemental Figure S4.

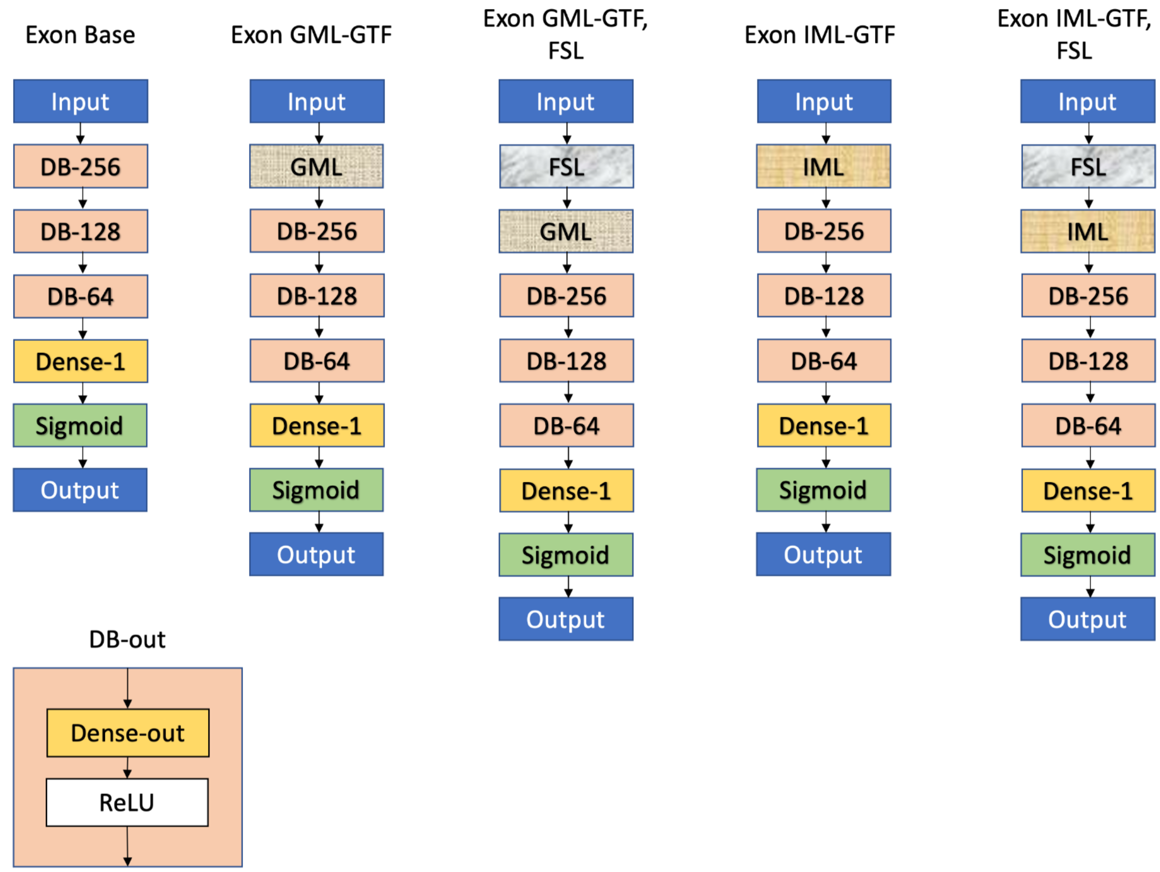

4) The author's comparison with cotinine-based classification of smoker status is an appropriate addition. However, it is not immediately clear what sort of model they used for the cotinine-based classification. I suspect it was a linear model, but this does not appear to be stated explicitly in the manuscript. Furthermore, it is worth noting that only 106 samples (i.e. ~21% of their test samples)

included plasma metabolite profiling of cotinine levels, so the authors should mention this limitation in their discussion.

Author response:
In our assessment of predictive accuracy of cotinine measures, ROC analysis was performed on measured cotinine values without further modeling or transformation. We have clarified this in the Methods, and we have specifically included the limitation of the relatively small test set sample size in the Discussion.

The text are updated accordingly:

Materials and methods (lines 190-191):

Predictive accuracy of serum cotinine for smoking status was assessed with ROC curves applied to measured cotinine values.

Discussion (lines 334-335):

However, cotinine values were only available for 106 of the test samples, and these observations may be sensitive to the method of cotinine measurement which was performed with SOMAscan technology in COPDGene.

5) The reanalysis with upper quartile (UQ) normalization appears to be sound, and represents a significant effort by the authors to remedy the issues I raised regarding their normalization procedure. However, it does not sufficiently address my concerns regarding the trimmed mean of M values (TMM) normalization using a reference set containing elements from their test set. I remain adamant that they should remove any results from any model trained on TMM-normalized data where the reference set includes examples from the test set. Although models trained with UQ-normalized data may produce similar performance metrics as those trained with TMM-normalized data (i.e. with test set information leakage), this is not the same as these models being the same. For instance, it is possible that the leakage of test set information into the TMM-based model will prevent it from generalizing well to new data. Given the sensitive issues and risks surrounding clinical classifiers, extra caution must be practiced here. Frankly, the authors should replace all TMM-based results (i.e. models, predictions, etc.) with those from UQ normalization, or simply re-apply TMM while explicitly using the set of training examples as TMM's reference set. Finally, in their discussion of the study's potential caveats, the authors should explicitly mention that their methods may not generalize to RNA-seq data generated with the more commonly used poly-A library selection method.

Author response:
We understand the reviewer's concern, and to address this we have followed their recommendation to remove our UQ normalized results and repeat our results such that all results come from TMM normalization where the reference sample always comes from the training dataset. The updated results are highlighted throughout the

text: see updated Table 2 and Table 3.

We have also added a sentence to the limitations section of the discussion regarding the fact that our results may not generalize to RNA-seq datasets generated with poly-A (rather than ribo-reduction only) protocols.

Discussion (lines: 354-356)
Our RNA-seq libraries were generated for total RNA with globin and ribosomal RNA reduction, thus our results may not translate directly to RNA-seq data generated with poly-A selection protocols.

Table 2. Predictive performance of modified Beineke models using gene, isoform and exon-level expression data.

	Val - Accuracy	Val - AUC	Test - Accuracy	Test - AUC
Gene	0.698	0.758	0.743	0.780
Isoform	0.757	0.827	0.774	0.828
Exon	0.801	0.859	0.808	0.869
Exon, GML-GTF	0.771	0.807	0.789	0.811
Exon, GML-GTF, FSL	0.776	0.805	0.741	0.796
Exon, IML-GTF	**0.828**	0.876	0.825	0.870
Exon, IML-GTF, FSL	**0.828**	**0.889**	**0.838**	**0.875**

Val: validation data. AUC: area under the receiver operating characteristic. IML-GTF: Isoform Map Layer containing information from Ensembl GTF file. GML-GTF: Gene Map Layer containing information from Ensembl GTF file. FSL: Feature Selection Layer. Best results are shown in bold.

Table 3. Predictive performance of various models using exon-level data, including elastic net for comparison.

	Val - Accuracy	Val - AUC	Test - Accuracy	Test - AUC
Exon, Elastic Net	0.821	0.861	0.774	0.903
Exon + Iso, Elastic Net	0.808	0.894	0.766	0.884
Exon Base	0.813	0.886	0.842	0.913
Exon, GML-GTF	0.833	0.899	0.842	0.913
Exon, GML-GTF, FSL	0.850	0.903	0.838	0.919
Exon, IML-GTF	0.843	0.905	0.854	0.924
Exon, IML-GTF, FSL	**0.860**	**0.916**	**0.869**	**0.935**

Val: validation data. AUC: area under the receiver operating characteristic. Exon + Iso: Concatenation of exon and isoform data. IML-GTF: Isoform Map Layer containing information from GTF file. GML-GTF: Gene Map Layer containing information from Ensembl GTF file. FSL: Feature Selection Layer. Best results are shown in bold.

6) This is a useful analysis, but it is not directly comparable to their primary results (i.e. Table 2) because of the differences in normalization procedures. The authors should use a single normalization method

throughout the manuscript, and then repeat this analysis. That being said, the removal of the Beineke model genes (which appears to have been done with a comparable normalization procedure) impacts model performance without totally abolishing it, which suggests that the authors gene set is a significant improvement over the five gene model and not just a rehashing of it.

Author response:
As indicated in the response to issue 5), all results in the main text now come from TMM-normalized data where the reference sample is always contained within the training dataset. The updated results for the 5 Beineke model genes with TMM normalization are now in the Supplemental Table S2. We agree with the reviewer's assessment that the sensitivity analysis in which our model performance is re-assessed by excluding the Beineke gene set indicates that our proposed model does include useful predictive information that is not contained solely within the five genes identified in the Beineke predictive model.

Table S2. **Predictive performance of modified Beineke models using gene, isoform and exon-level expression data, with *MUC1* included and TMM normalized as in the main paper.**

	Val - Accuracy	Val - AUC	Test - Accuracy	Test - AUC
Gene	0.668	0.651	0.669	0.666
Isoform	0.752	0.813	0.758	0.815
Exon	0.806	0.86	0.782	0.855
Exon, IML-GTF	**0.830**	0.876	0.821	0.871
Exon, IML-GTF, FSL	**0.830**	**0.892**	**0.834**	**0.875**

Val: validation data. AUC: area under the receiver operating characteristic. IML-GTF: Isoform Map Layer containing information from Ensembl GTF file. FSL: Feature Selection Layer. Best results are shown in bold.

7) My point was more concerned with the general incompleteness of commonly-used annotation sets of splice sites and isoforms rather than a comparison between ENSEMBL and GENCODE in specific. For instance, did the authors consider using novel splice sites from a database like Snaptron or even simply a large RNA-seq study like GTEx? Although such catalogs may be noisey, they may also include isoforms that are useful for this study. The authors need not repeat their analysis with such a set, but it could be worth mentioning as a potential future improvement.

Author response:
We understand and concur with this point. We have added a sentence to the limitations portion of our discussion that reads as follows:

Discussion (lines 357-360):

While our study expands upon gene-level quantification studies by demonstrating the additional utility of exon-level quantification, future improvements might be obtained by considering novel, unannotated isoforms and exon

junctions, rather than restricting to known transcript models as we have done here.

8) This addition is an improvement and more clearly conveys the motivation behind the Feature Selection Layer (FSL). Could the authors explain in more detail why they expected the L1 constraint to improve generalizability or provide a citation supporting this notion?

Author response:
First of all, L1 constraint, as a regularization technique, improves model generalizability (see https://en.wikipedia.org/wiki/Regularization_(mathematics)) Moreover, L1 constraint encourages sparsity of the weights of FSL (see https://www.stat.cmu.edu/~ryantibs/statml/lectures/sparsity.pdf), meaning that the feature importance scores will be close to 0 or 1, which helps us distinguish between important and unimportant features more easily.

9) The addition of the "Model Interpretation" section is a significant improvement in the manuscript. I would like more analysis in this direction however. For instance, how many of these genes are highly expressed in the lung or differentially expressed in smokers? Are there any isoforms or exons that appear important and are known to be specifically associated with smoking status or related phenotypes? In general, it would be nice to see if this model recapitulates known associations, and also whether it can be used to identify novel biology. Further analysis here would also greatly improve the demonstrated biological relevance of this work, and also provide and describe potential avenues for future biological research.

Author response:
Some clarification regarding the exons with high saliency scores is required - namely, in our prediction models we considered only genes in the set of 1270 that had been previously associated with smoking in the gene-level analysis by Huan et al (lines 220-222). Thus, all of the genes and exons considered for our models have been shown previously to be associated with smoking status, and so by definition the important genes from our exon-level model recapitulate known associations from prior gene-level analyses which is why we did not perform any overlap analysis with prior smoking-associated genes. Considering that our background set of genes has previously been associated with smoking, this clarifies how our enrichment analysis based on saliency map importance scores provides a different perspective than enrichment analyses done in previous smoking studies (as described in our response to issue 1 for reviewer 2).

To address the issue of overlap between genes containing important exons for prediction, we referred back to the Huan et al. paper which identified 30 of their 1270 significant genes that had been identified in any one of three previous smoking differential gene expression studies in lung tissue. Our top 20% of important exons map to 586 genes, and 16 of the 30 "lung overlap" genes from the Huan study are present in these 586 genes, which is not a significant enrichment. Given that this analysis was non-informative we did not include this in the manuscript.

10) The authors have almost answered all of my questions about the training procedure. However, could they provide additional information regarding their early stopping strategy? In specific, what were their criteria for early stopping?

<u>Author response:</u>
Thanks for this question, we stop training and get the best performing model when there is no increase in validation accuracy for 10 epochs.

We have also added the criteria for early stopping in our manuscript:

Materials and methods (lines 125-127):
We employ the early stopping strategy during training. Specifically, we stop training and get the best performing model on the validation set so far when there is no increase in validation accuracy for 10 epochs.

11) The authors have addressed my concerns regarding the readability of their uploaded figures.

<u>Minor Points</u>
All of the minor points have been sufficiently addressed.

Reviewer 2:

<u>Minor Points</u>

1. The additional work on model interpretation strengthens this manuscript. However, the biological findings (e.g., top pathways related to GTPase activity and protein ubiquitination/degradation) are not connected to any of the findings from prior related work. It would be helpful if the authors could comment on whether these findings reinforce prior findings or are novel by citing prior work.

<u>Author response:</u>

The enriched pathways identified from our importance analysis of exon features did not have much overlap with pathways identified in two published gene-level smoking analyses by Parker et al. and Huan et al. We included this in a new paragraph in the Results section, and we also describe how the difference in the two enrichment analyses is somewhat expected since the nature of the enrichment analyses in this study is different from the prior two studies. This explanation reads as follows:

Results (line 266-274):

The most enriched pathways in these studies were primarily related to immune response, wound healing, and

platelet activation. This difference is expected since the enrichment analyses performed for this study was geared to identify genes whose exon-level information was important for predicting smoking status relative to our background set of genes which consisted of the smoking-associated genes from the gene-level study of Huan et al.

2. It is excellent that the authors have made all of the code and supporting files available. However, some of the key files (e.g., the isoform-exon maps and network weights) are only available via a Google drive link. Files in Google Drive can be easily inadvertently moved or deleted. I would strongly suggest archiving and versioning these key files on a site such as Zenodo to ensure reproducibility of this work well into the future.

Author response:

We appreciate this suggestion. We have uploaded our code as well as key files to Zenodo, with DOI 10.5281/zenodo.5136729 and corresponding link https://doi.org/10.5281/zenodo.5136729

We also updated the corresponding text in our manuscript:

Materials and methods (lines 132-134):

All network definitions, network weights and code, as well as additional files required for reproducing our experiment results are available at https://doi.org/10.5281/zenodo.5136729.
