# Supplementary material for: Improved prediction of smoking status via isoform-aware RNA-seq deep learning models
Source: PLoS Comput Biol. 2021 Oct 11;17(10):e1009433. doi: 10.1371/journal.pcbi.1009433 (PMC8530282; doi:10.1371/journal.pcbi.1009433)
Supplement: Attachment — Submitted filename: reviewer_comments_revision_2.pdf [file pcbi.1009433.s011.pdf]

General Opinion:

The authors have addressed most of my concerns with the manuscript. Aside from one minor addition regarding their L1 regularization in the face of collinear features, I feel that the manuscript has reached an acceptable state.

Major Points:

1) By uploading their code to Zenodo, the authors have addressed my concerns with the future accessibility of their materials.
2) The new figure more clearly conveys the neural network architectures used in the paper.
3) The authors have sufficiently addressed my comments regarding the cotinine-based model.
4) The authors have fixed the issues with the TMM-based normalization.
5) The authors have addressed my concerns with their use of two normalization procedures.
6) The manuscript now clearly conveys the limitations of the splicing annotations used.
7) Perhaps the reviewers could simply state that regularization will reduce overfitting, thereby enhancing the models generalizability. Moreover, it is worth noting that L1 regularization - as an interpretability technique - is confounded when the number of features exceeds the number of samples or when there are collinear features.
8) The authors have adequately addressed my concerns regarding model interpretation.
9) The authors have addressed my questions regarding early stopping.
